# Management and Outcomes of Sternoclavicular Joint Infections: A Retrospective Study

**DOI:** 10.3390/jcm14061893

**Published:** 2025-03-11

**Authors:** Edin Ahmic, Paul Swatek, Iurii Mykoliuk, Anton Busau, Paul Bamberg, Josef Smolle, Freyja Maria Smolle-Juettner, Jörg Lindenmann

**Affiliations:** 1 Division of Thoracic and Hyperbaric Surgery, Department of Surgery, Medical University of Graz, 8036 Graz, Austria; paul.swatek@medunigraz.at (P.S.); iurii.mykoliuk@medunigraz.at (I.M.); anton.busau@medunigraz.at (A.B.); paul.bamberg@stud.medunigraz.at (P.B.); freyja.smolle@medunigraz.at (F.M.S.-J.); jo.lindenmann@medunigraz.at (J.L.); 2 Institute of Medical Informatics, Statistics and Documentation, Medical University of Graz, 8036 Graz, Austria; josef.smolle@medunigraz.at

**Keywords:** sternoclavicular joint infection, muscle flap reconstruction, chest wall, negative pressure wound therapy, sternum infection

## Abstract

**Introduction**: Sternoclavicular joint infections (SCJIs) are extremely rare, making up less than 1% of all septic arthritis cases. This retrospective study aims to evaluate the management and outcomes of SCJIs, including both surgical and non-surgical approaches. **Methods**: This retrospective study included 55 patients treated between January 2005 and December 2023 at the Division of Thoracic and Hyperbaric Surgery in Graz, Austria. Data on patient characteristics, treatment approach, and outcome were analyzed. **Results**: Out of the 55 patients, 50 (90.91%) underwent surgery. Among them, 21 (38.18%) had pleural involvement and 9 (16.36%) developed sepsis. Primary debridement and sternoclavicular joint resection with muscle flap closure were performed in 5 patients (9.3%), whilst debridement and negative pressure wound therapy (NPWT) followed by joint resection were applied in 32 patients (59.3%). In total, 15 (27.2%) of these cases required a secondary muscle flap. Positive cultures were found in 35 patients (63.64%), with Staphylococcus aureus being the most common pathogen. Multivariate analysis identified elevated CRP and leukocyte levels as significant predictors of sepsis. Defects requiring myoplastic procedures were associated with a higher risk of complications, sepsis, and prolonged hospital stays. Postoperative complications occurred in 20 patients (36.36%), but there was no 30-day mortality. **Conclusions**: SCJI is a rare but serious condition that requires prompt surgical intervention. Our findings suggest that combining surgical resection with NPWT and/or myocutaneous flap techniques is effective. Close monitoring of inflammatory markers is crucial for identifying sepsis risk and improving patient outcomes.

## 1. Introduction

Sternoclavicular joint infections (SCJIs) are extremely rare, representing less than 1% of all septic arthritis cases, and their true incidence remains largely unknown [1,2,3,4,5]. While surgical treatment, particularly resection of the joint, has been highlighted as a definitive option for cases unresponsive to antibiotic therapy [4], recent studies suggest that targeted antibiotic therapy may suffice for selected patients [5]. However, when infections significantly affect the bone, adjacent tissues, pose a risk of sepsis, or result in periarticular fluid accumulation, surgical intervention is often preferred [6,7]. En bloc resection is considered the gold standard, as it offers superior outcomes compared to standard debridement by fully removing the infected area. Negative pressure wound therapy (NPWT) is used for several weeks to promote granulation, depending on the severity and extent of the infection [8]. For larger wounds, reconstruction using muscle flaps, such as the pectoralis major flap, is recommended [9]. Other viable options include alternative muscle flaps, such as the latissimus dorsi [10,11,12].

Signs of soft tissue infection near the joint, such as swelling, streaking, or abscess formation, may indicate joint involvement but must be differentiated from actual joint infections. Typically, soft tissue infections respond well to antibiotic treatment alone. However, joint infections or abscesses require drainage and/or debridement. Imaging that reveals osteomyelitis in the clavicle, manubrium, or first rib strongly suggests the need for joint resection or thorough debridement, rather than relying solely on incision, drainage, or antibiotic therapy [6].

This retrospective study aims to evaluate the management and outcomes of SCJIs, including both surgical and non-surgical approaches. Given the rarity and complexity of SCJIs, we assessed different treatment strategies, including debridement with NPWT, joint resection with muscle flap closure, and conservative antibiotic therapy in selected cases.

## 2. Methods

This retrospective study was conducted at a tertiary university-based care center. Patient records from January 2005 till December 2023 were reviewed. The Medical University of Graz Ethics Committee granted ethical approval for this study (EK: 28-015 ex 15/16, approved on 6 October 2015).

### 2.1. Diagnostic Procedures

**Figure 1:**
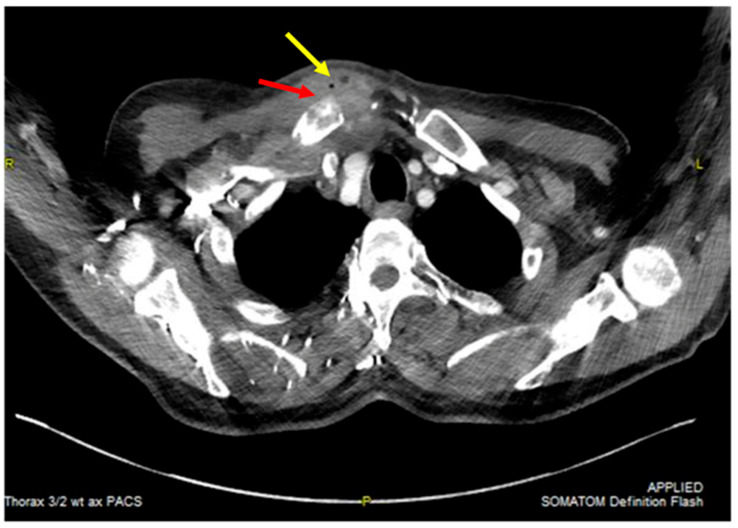
Axial CT scan of the chest showing an abscess in the right sternoclavicular joint region with expansion into adjacent soft tissues. Air inclusions (indicated by the yellow arrow) are visible, along with signs of osteomyelitis in the surrounding bone structures (indicated by the red arrow), indicating an advanced stage of infection.

Routine diagnostic procedures included physical examination, measurement of body temperature, laboratory findings (white blood-cell count, CRP, renal and hepatic function parameters, as well as blood cultures in presence of fever), chest roentgenogram, and computed tomography (CT-scan) of the chest. If osteomyelitis could not be ruled out by the latter, magnetic resonance imaging (MRI) was used. Figure 1 shows an axial CT scan of the chest, indicating an abscess in the sternoclavicular joint area that extends into the surrounding soft tissues. Thickened and inflamed tissue suggests an advanced stage of infection.

### 2.2. Surgical Technique

**Figure 2:**
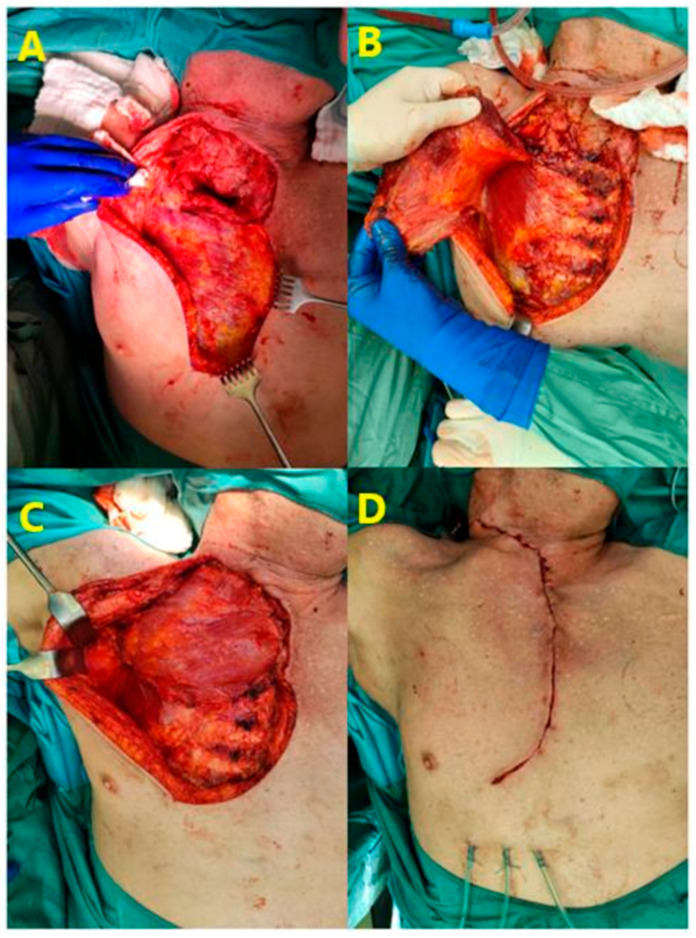
Intraoperative images showing the use of the pectoralis major muscle flap for closure of the defect following resection of the sternoclavicular joint. The upper images illustrate the mobilization of the flap, with (**A**) showing granulation tissue in the area of the sternoclavicular joint after negative pressure wound therapy (NPWT) and mobilization of the skin and (**B**) depicting the mobilization of the pectoralis major muscle. The lower images display the final positioning of the flap, where (**C**) shows the placement of the muscle over the defect and (**D**) demonstrates the layered closure of the wound, including muscular, subcutaneous, and intradermal suturing.

An incision was made directly over the affected sternoclavicular joint (SCJ), the abscess was opened and a swab for microbiological analysis was taken. Debridement of abscess membrane and necrotic tissue usually included subcutaneous structures, the removal of the sternal and clavicular portions of the sternocleidomastoid muscle and in single cases the medial portion of the pectoralis minor muscle. Depending on the local findings, the sternoclavicular joint was resected initially or after primary conditioning by negative wound pressure therapy (NPWT). The sponges were changed every three to four days until clean granulative tissue had developed. Depending on the size of the defect direct wound closure or closure by a pedicled pectoralis major muscle flap was carried out. Figure 2 demonstrates the application of a pectoralis major muscle flap to cover a large defect following extensive debridement of infected tissue. If the initial debridement had led to clean surfaces, one-stage closure was possible. Myoplastic procedures were categorized into primary and secondary muscle flap closures. Primary one-stage closure was performed in 5 patients (9.3%), while secondary closure after NPWT was required in 15 patients (27.2%). In all other cases NPWT preceded definitive wound closure. Hyperbaric oxygen (HBO) therapy was applied as an adjunct treatment to promote healing in the presence of osteomyelitis. Antibiotic administration was tailored to each patient, based on inflammatory markers, infection signs, and microbiological findings from swabs or blood cultures. In presence of pleural effusion, a chest-tube drainage was inserted.

### 2.3. Statistical Analysis

Statistical analysis was performed to evaluate both categorical and continuous variables. Descriptive statistics were used to summarize the data, with continuous variables presented as means and standard deviations, while categorical variables were expressed as frequencies and percentages. The analysis was performed using Stata 17 (StataCorp LLC, College Station, TX, USA).

For comparisons between groups, a t-test was applied to continuous variables and the chi-square test was used for categorical variables. To assess independent risk factors for sepsis, complications, and length of hospital stay, a multivariate logistic regression analysis was conducted. The dependent variables included sepsis, complications, and hospital stay duration. Independent variables considered in the model included age, gender, pleural involvement, presence of pathogens, obesity, diabetes mellitus, intravenous drug use, hypertension, renal insufficiency, pain, swelling, fever, C-reactive protein (CRP) levels, and leukocyte count. NPWT and myoplastic procedures were applied depending on the clinical presentation.

Odds ratios (ORs) and their corresponding 95% confidence intervals (CIs) were calculated to assess the strength of the associations between the independent variables and the outcomes. Statistical significance was set at a *p*-value of less than 0.05.

## 3. Results

A total of 55 patients (35 men [63.64%] 20 women [36.36%], mean age: 65.31 years [±12.82 years]) treated at the Division of Thoracic and Hyperbaric Surgery, at the Medical University Graz between January 2005 and December 2023 were included in the study. Comorbidities and risk factors such as obesity, diabetes mellitus, renal insufficiency, i.v. drug abuse, or immunodeficiency were present in 37 (67.2%) patients (see Table 1). In addition to demographic and laboratory data, a detailed clinical history was reviewed, including prior trauma, previous infections, history of contact sports participation, recent arthrocentesis, and past surgical procedures involving the thoracic region or upper extremities. These factors were considered to assess potential predisposing conditions for SCJI.

Swelling (*n* = 48; 87.2%) with or without erythema, pain (*n* = 39; 70.9%) and fever (*n* = 18; 32.7%) were the predominant symptoms at the time of admission. Fifty out of the fifty-five patients (90.91%) underwent surgery; five patients without radiologically confirmed bone involvement or abscess were treated with intravenous antibiotics alone.

Of the 50 patients (90.91%) who underwent surgery, 9 presented with sepsis. In total, 21 patients (38.18%) had intrathoracic involvement with subpleural extension of abscess that caused pleural effusion in 14 instances. Intraoperative swabs yielded positive microbiological findings in 35 patients (63.64%), most frequently Staphylococcus aureus in 15 patients (27%) and Staphylococcus epidermidis in 5 patients (9%). Additional pathogens isolated in single patients included *Cutibacterium acnes*, *Streptococcus agalactiae*, *Staphylococcus schleiferi*, *Staphylococcus warneri*, Methicillin-resistant *Staphylococcus aureus* (MRSA), *Streptococcus canis*, *Enterobacter cloacae* complex, *Staphylococcus haemolyticus*, and *Streptococcus pneumoniae*. Among the 50 surgically treated patients, 35 (63.64%) had positive intraoperative cultures, with *Staphylococcus aureus* being the most frequent isolate. The high proportion of culture-negative cases (36.36%) may be attributed to prior antibiotic therapy, which was administered in all 50 patients before surgery. Primary one-stage debridement, sternoclavicular joint resection, and muscle flap closure was carried out in five patients (9.3%). Debridement of the abscess and NPWT with secondary resection of the sternoclavicular joint were carried out in 32 patients (59.3%) and conditioning and shrinking of the defect notwithstanding, secondary muscle flap closure was required in 15 patients (27.2%) after a median of 3.09 NPWT changes (range 1–12). In addition, eight patients (14.55%) received HBO therapy to support healing in the presence of complex inflammation extending into the clavicle and the sternum adjacent to the resected area. In total, 14 patients had a chest tube for pleural effusion (see Table 2).

Postoperative complications occurred in 20 patients (36.36%). There were three cases of subpectoral hematoma, two cases of seroma, one recurrent abscess, and one fistula, all requiring surgical intervention. Two patients developed pleural empyema in spite of chest-tube drainage and required decortication. Eight patients developed postoperative pleural effusion requiring thoracic drainage without decortication. Two cases of anterior mediastinitis were managed by antibiotic treatment, one patient had severe cutaneous drug reaction. There was no 30-day mortality. The median hospital stay was 20.75 days (±12.48), with a range of 7 to 40 days.

To define factors associated with sepsis, postoperative complications, and the length of hospital stay, we conducted a multivariable analysis. The variables analyzed included gender, age, pleural involvement, the use of NPWT, myoplastic procedures, the presence of pathogens, comorbidities in general, obesity, diabetes mellitus, intravenous drug use, renal insufficiency, pain, swelling, fever, C-reactive protein (CRP), and white blood cell count (WBC).

Notably, the need for myoplastic procedures was significantly more likely in cases associated with sepsis (OR 4.57, *p* = 0.050) and complications (OR 3.53, *p* = 0.03), and also contributed to a longer hospital stay (coefficient 8.41, *p* = 0.015). Additionally, pleural involvement was linked with increased odds of sepsis (OR 2.34, *p* = 0.249) and a longer hospital stay (coefficient 3.33, *p* = 0.34), although these results were not statistically significant.

Both CRP levels (OR 1.01, *p* = 0.003) and leukocyte count (OR 1.16, *p* = 0.004) were significant predictors of sepsis. Higher CRP and WBC levels were associated with increased odds of sepsis. Fever was also a significant predictor of complications (OR 3.37, *p* = 0.04) and was associated with a longer hospital stay (coefficient 7.48, *p* = 0.036).

Other factors, such as NPWT (coefficient 3.96, *p* = 0.00) and pathogen presence (OR 2.25, *p* = 0.344), were associated with a longer hospital stay but did not reach statistical significance for sepsis or complications (see Table 3).

## 4. Discussion

SCJI is a rare but serious condition that can lead to systemic complications, with known risk factors such as diabetes mellitus, immunodeficiency, advanced renal disease, intravenous drug use, trauma, or central venous catheters [6]. In our study, the most prominent risk factors included diabetes mellitus (32.73%), renal insufficiency (16.36%), and intravenous drug use (5.45%). No patients had a history of intra-articular injections, central venous catheters, or rheumatoid arthritis, although one fourth was obese with a BMI > 30.

We can confirm, as stated in the review by Ross and Shamsuddin, that these predisposing risk factors do not exist in many patients. In their analysis, they reviewed literature on SCJI from 1970 to 2003, covering 170 reported cases. They found that 23% of these cases had no predisposing conditions, 21% were associated with intravenous drug use, 24% involved a distant infection including those related to central lines, and 13% were linked to diabetes. Other less frequent conditions included chronic renal failure, alcoholism, cirrhosis, and HIV infection [13].

Five of our patients were sufficiently treated by antibiotic therapy alone, as they had neither sepsis nor radiological signs of abscess or osteomyelitis. In addition to intravenous antibiotics, which are considered as first line treatment in SCJI, we carried out surgery in 50 of the 55 patients (90.91%) [14]. This is in accordance with the literature where surgical treatment is recommended in SCJI with periarticular fluid collection, or abscesses and bone involvement [7]. In all our surgically treated cases debridement involved the resection of the sternoclavicular joint. Our findings align with Abu Arab et al., who demonstrated that surgical resection is a curative option for SCJI in patients unresponsive to antibiotic therapy [6]. However, unlike their cohort, where complex muscle flap reconstructions were rarely necessary, our study identified a significant need for secondary muscle flap closures in 27.2% of cases, highlighting the complexity of defects in advanced infections. As Kachala et al. point out, sternoclavicular joint resection can be performed with minimal or no impact on upper extremity function, provided that the costoclavicular ligament remains intact [15]. This principle was followed in our cases to ensure postoperative functional outcomes.

According to Schreiner et al., the combination of negative pressure wound treatment (NPWT) with instillation and dwell time (NPWTdt) improves bacterial eradication and shortens the duration of wound care. We used NPWT in 32 patients (59.3%), although NPWTdt was not utilized. The median hospital stay for our patients was 20.75 days, while the literature reports that patients treated with NPWTdt have a median hospital stay of 25 days [16]. For patients with complex defects, hyperbaric oxygen (HBO) therapy was employed to support wound healing, as described in a case report by Tanaka et al., where HBO therapy was successfully used to close a complex wound in a patient with SCJI [17].

In total, 36.36% of our patients required myoplastic procedures for closure of large defects, particularly the use of pectoralis major muscle flaps. According to Barkat et al. the rectus abdominis muscle flap may serve as a viable alternative if the pectoralis major and latissimus dorsi muscles be unavailable. We did not have to resort to using this option even in case of redo-surgery where the original flap could be preserved and re-positioned [18].

The rate of complications reported in the literature varies widely, from 0% (Jang et al.) [19] to 40% (Bakaeen et al. and Puri et al.) and is in part depending on its definition [20,21]. In our study, postoperative complications developed in 20 patients (36.36%). Of note, recurrence of abscess occurred in three patients (5.6%) requiring redo surgeries, which included re-debridement and repositioning of the previously dissected myocutaneous flap. Recurrence of osteomyelitis was noted in the following studies, with rates ranging from 6% (Ali et al.) [22] and 10% (Kachala et al. and Ross et al.) [13,15] to 23% (Von Glinksi et al.) [23]. We observed no mortality within the first 30 days. No cases of osteomyelitis were observed in our cohort, as confirmed by imaging and intraoperative findings. Consistent with the study by Pothini et al., indicators such as significant abscess size and elevated inflammatory markers were associated with poorer outcomes [5]. Our multivariate analysis further confirmed CRP and leukocyte count as significant predictors of sepsis, underscoring the importance of close monitoring in high-risk patients.

SCJI is primarily attributed to hematogenous spread, but direct extension from adjacent infections, trauma, or invasive procedures (e.g., arthrocentesis, central venous catheters) may also contribute [6,13]. In our cohort, Staphylococcus aureus was the most frequently isolated pathogen, followed by Staphylococcus epidermidis. Cutibacterium acnes was found in only one case, indicating a minor role in SCJI. While C. acnes is frequently isolated in shoulder surgery, where it is associated with postoperative infections and implant-related complications, its clinical significance in SCJI appears to be limited [24,25,26].

Our study identified several factors associated with sepsis, complications and extended hospital stays. The multivariable analysis showed that there was a significant correlation between both sepsis (OR 4.57, *p* = 0.050) and complications (OR 3.53, *p* = 0.03) with myoplastic procedures, indicating that in these instances the defects tended to be larger. Obviously, CRP level (OR 1.01, *p* = 0.003) and leukocyte count (OR 1.16, *p* = 0.004) were significant predictors of sepsis, confirming the role of inflammatory markers in identifying patients at risk for systemic infection.

Interestingly, although pleural involvement was associated with an increased risk of sepsis and a longer hospital stay, these findings did not reach statistical significance. However, pleural involvement remains clinically relevant, as it indicates a more advanced stage of infection, which may necessitate more aggressive surgical management as was found in two of our patients who required pleural decortication.

The median length of hospital stay was 20.75 days (±12.48). Factors such as the need for NPWT (coefficient 3.96, *p* = 0.00) and the presence of fever (coefficient 7.48, *p* = 0.036) were associated with prolonged hospitalization, likely reflecting the severity of the infection and the need for extended wound care. The use of NPWT, in particular, has been shown to promote faster granulation of large defects enabling definitive wound closure, although it often requires multiple changes of sponges, contributing to a longer overall hospital stay.

One limitation of our study is the selection bias, as only hospital-treated cases were included, potentially leading to an overrepresentation of more severe SCJI cases. Additionally, due to the retrospective nature of the study, structured long-term follow-up data were not available. However, given the rarity of SCJI, our cohort of 55 patients over nearly two decades represents a valuable dataset for single-center evaluation. However, sternoclavicular joint infection is a rare disease and a cohort of 55 patients treated within almost 20 years represents a considerable sample for a single-center evaluation. Future multicenter studies should be directed at exploring additional treatment options for managing SCJI.

## 5. Conclusions

Sternoclavicular joint infections are rare and can lead to serious complications, including sepsis and prolonged hospitalization. Based on our findings, debridement including resection of the sternoclavicular joint combined with NPWT and/or myocutaneous flap closure has proven effective in treating these infections. Our multivariate analysis showed that elevated CRP and leukocyte levels were significant predictors of sepsis. Larger defects eventually requiring myoplastic closure were significantly correlated with sepsis, with the development of complications and with a prolonged hospital stay. A multidisciplinary approach, including close monitoring of inflammatory markers and comprehensive wound care, is essential for reducing complications and improving patient outcomes. Continuing research is necessary to optimize treatment protocols and explore additional therapeutic options for these patients.

## Figures and Tables

**Table 1:** Comorbidities and risk factors (*n* = 55).

Variable	Number (%)
Comorbidities total	37 (67.27)
Obesity	14 (25.45)
Diabetes Mellitus	9 (16.36)
Renal Insufficiency	8 (14.55)
Immunodeficiency	2 (3.6)
I.V. Drug Abuse	3 (5.45)

**Table 2:** Clinical findings of patients with sternoclavicular joint infection (*n* = 55).

Variable	Number (%)
Positive blood cultures	36 (65.45)
Sepsis	9 (16.36)
Pleural involvement	21 (38.18)
Primary joint resection and flap closure	5 (9.09)
Secondary flap closure	15 (27.27)
Total number of flap closure	20 (36.36)
Hyperbaric oxygen treatment	8 (14.54)
Postoperative complications	20 (36.36)
Recurrence	3 (5.45)
30-day mortality	0 (0)
NPWT changes [Mean (Std. Dev.)]	3.09 [2.55]
Length of hospital stay [Mean (Std. Dev.)]	20.75 [12.48]

**Table 3:** Multivariable analysis of factors associated with sepsis, complications, and length of hospital stay.

Variable	Sepsis (OR)	Sepsis (95% CI)	*p*-Value	Complications (OR)	Complications (95% CI)	*p*-Value	Hospital Stay (Coefficient)	*p*-Value
Gender (Male)	1.17	0.97–1.41	0.836	1.09	0.94–1.27	0.87	−3.93	0.26
Age	1.04	0.47–2.28	0.232	0.99	0.81–1.22	0.82	0.42	0.10
Pleural involvement	2.34	1.09–5.04	0.249	1.56	0.87–2.79	0.43	3.33	0.34
NPWT	1.10	0.63–1.91	0.459	1.22	1.13–1.31	0.94	3.96	0.00
Myoplastic procedure	4.57	1.54–13.5	0.050	3.53	1.11–11.19	0.03	8.41	0.015
HBO therapy	-	-	-	1.05	0.98–1.13	0.94	−2.33	0.62
Pathogen (positive)	2.25	1.16–4.38	0.344	2.25	0.97–5.2	0.19	4.23	0.22
Comorbidities	0.54	0.3–0.98	0.410	1.77	0.91–3.42	0.35	0.28	0.93
Obesity	1.03	0.99–1.07	0.966	1.17	0.91–1.5	0.78	1.53	0.67
Diabetes mellitus	0.21	0.09–0.5	0.161	2.36	0.95–5.84	0.14	−1.52	0.67
Renal insufficiency	1.59	1.05–2.41	0.606	0.44	0.23–0.86	0.34	0.43	0.92
I.V. drug abuse	-	-	-	3.77	1.83–7.76	0.29	−5.72	0.44
Pain	1.53	1.02–2.28	0.622	2.08	0.98–4.42	0.26	2.02	0.59
Swelling	1.20	1.03–1.4	0.870	1.50	1.02–2.2	0.64	−4.71	0.35
Fever	3.17	1.24–8.1	0.120	3.37	1.1–10.29	0.04	7.48	0.036
CRP	1.01	0.25–4.01	0.003	1.00	0.45–2.23	0.22	0.01	0.23
WBC	1.16	0.3–4.5	0.004	0.95	0.45–2.0	0.27	0.02	0.90

## Data Availability

The data underlying this article will be shared on reasonable request to the corresponding author.

## References

[1] Edwin J., Ahmed S., Verma S., Tytherleigh-Strong G., Karuppaiah K., Sinha J. (2018). Swellings of the sternoclavicular joint: Review of traumatic and non-traumatic pathologies. EFORT Open Rev..

[2] McAninch S.A., Smithson C., Juergens A.L., Collins J.N., Nanda A. (2018). Sternoclavicular Joint Infection Presenting as Nonspecific Chest Pain. J. Emerg. Med..

[3] Thompson M.A., Barlotta K.S. (2018). Septic Arthritis of the Sternoclavicular Joint. J. Emerg. Med..

[4] Abu Arab W., Khadragui I., Echavé V., Deshaies A., Sirois C., Sirois M. (2011). Surgical management of sternoclavicular joint infection. Eur. J. Cardio-Thorac. Surg..

[5] Pothini T., Wilmot C.D., Waters J.K., Wait M.A., Reznik S.I., Jordan K.G., Caire J.T., Ashworth J.M., Cady L.C., Lysikowski J.R. (2024). Clinical and radiological septic joint analysis of spontaneous sternoclavicular joint infections: Achieving the best outcomes—A systems engineering approach. Eur. J. Cardio-Thorac. Surg..

[6] Tasnim S., Shirafkan A., Okereke I. (2020). Diagnosis and management of sternoclavicular joint infections: A literature review. J. Thorac. Dis..

[7] Nusselt T., Klinger H.M., Freche S., Schultz W., Baums M.H. (2011). Surgical management of sternoclavicular septic arthritis. Arch. Orthop. Trauma. Surg..

[8] Joethy J., Lim C.H., Koong H.N., Tan B.K. (2012). Sternoclavicular joint infection: Classification of resection defects and reconstructive algorithm. Arch. Plast. Surg..

[9] Al-Mufarrej F., Martinez-Jorge J., Carlsen B.T., Saint-Cyr M., Moran S.L., Mardini S. (2013). Use of the deltoid branch-based clavicular head of pectoralis major muscle flap in isolated sternoclavicular infections. J. Plast. Reconstr. Aesthet. Surg..

[10] Raymond D. (2014). Surgical intervention for thoracic infections. Surg. Clin. N. Am..

[11] Chen H., Ji X., Hao M., Zhang Q., Tang P. (2016). A three-stage procedure using bone transportation for the treatment of sternoclavicular infectious arthritis. J. Orthop. Surg. Res..

[12] Bendon C.L., Giele H.P. (2014). Second toe metatarsophalangeal joint transfer for sternoclavicular joint reconstruction. J. Hand Surg. Am..

[13] Ross J.J., Shamsuddin H. (2004). Sternoclavicular septic arthritis: Review of 180 cases. Medicine.

[14] Murga A., Copeland H., Hargrove R., Wallen J.M., Zaheer S. (2017). Treatment for sternoclavicular joint infections: A multi-institutional study. J. Thorac. Dis..

[15] Kachala S.S., D’Souza D.M., Teixeira-Johnson L., Murthy S.C., Raja S., Blackstone E.H., Raymond D.P. (2016). Surgical Management of Sternoclavicular Joint Infections. Ann. Thorac. Surg..

[16] Schreiner W., Ludolph I., Dudek W., Horch R.E., Sirbu H. (2020). Negative Pressure Wound Therapy Combined with Instillation for Sternoclavicular Joint Infection. Ann. Thorac. Surg..

[17] Tanaka Y., Kato H., Shirai K., Nakajima Y., Yamada N., Okada H., Yoshida T., Toyoda I., Ogura S. (2016). Sternoclavicular joint septic arthritis with chest wall abscess in a healthy adult: A case report. J. Med. Case Rep..

[18] Ali B., Petersen T.R., Shetty A., Demas C., Schwartz J.D. (2021). Muscle flaps for sternoclavicular joint septic arthritis. J. Plast. Surg. Hand Surg..

[19] Jang Y.R., Kim T., Kim M.C., Sup Sung H., Kim M.N., Kim M.J., Kim S.H., Lee S.O., Choi S.H., Woo J.H. (2019). Sternoclavicular septic arthritis caused by *Staphylococcus aureus*: Excellent results from medical treatment and limited surgery. Infect. Dis..

[20] Puri V., Meyers B.F., Kreisel D., Patterson G.A., Crabtree T.D., Battafarano R.J., Krupnick A.S. (2011). Sternoclavicular joint infection: A comparison of two surgical approaches. Ann. Thorac. Surg..

[21] Bakaeen F.G., Huh J., Fagan S.P., Bellows C.F. (2008). Surgical treatment of sternoclavicular joint infections in cirrhotic patients. Am. J. Surg..

[22] Ali B., Shetty A., Qeadan F., Demas C., Schwartz J.D. (2020). Sternoclavicular Joint Infections: Improved Outcomes with Myocutaneous Flaps. Semin. Thorac. Cardiovasc. Surg..

[23] von Glinski A., Yilmaz E., Rausch V., Koenigshausen M., Schildhauer T.A., Seybold D., Geßmann J. (2019). Surgical management of sternoclavicular joint septic arthritis. J. Clin. Orthop. Trauma..

[24] Chuang M.J., Jancosko J.J., Mendoza V., Nottage W.M. (2015). The Incidence of Propionibacterium acnes in Shoulder Arthroscopy. Arthroscopy.

[25] Mook W.R., Klement M.R., Green C.L., Hazen K.C., Garrigues G.E. (2015). The Incidence of Propionibacterium acnes in Open Shoulder Surgery: A Controlled Diagnostic Study. J. Bone Joint Surg. Am..

[26] Phadnis J., Gordon D., Krishnan J., Bain G.I. (2016). Frequent isolation of Propionibacterium acnes from the shoulder dermis despite skin preparation and prophylactic antibiotics. J. Shoulder Elbow Surg..

